# *N*,*N*′-Bis(2-cyano­ethyl)-4,4′-dimethyl-*N*,*N*′-(butane-1,4-di­yl)dibenzene­sulfonamide

**DOI:** 10.1107/S1600536809036022

**Published:** 2009-09-12

**Authors:** Peng-Fei Cheng, Chao-Jie Wang, Yu-Xia Wang

**Affiliations:** a College of Chemistry and Chemical Engineering, Henan University, Kaifeng 475004, People’s Republic of China

## Abstract

The complete mol­ecule of the title compound, C_24_H_30_N_4_O_4_S_2_, is generated by a crystallographic inversion centre. In the crystal, weak C—H⋯O inter­actions link the mol­ecules, forming infinite sheets.

## Related literature

For background to polyamines, see: Thomas & Thomas (2003 ▶). 
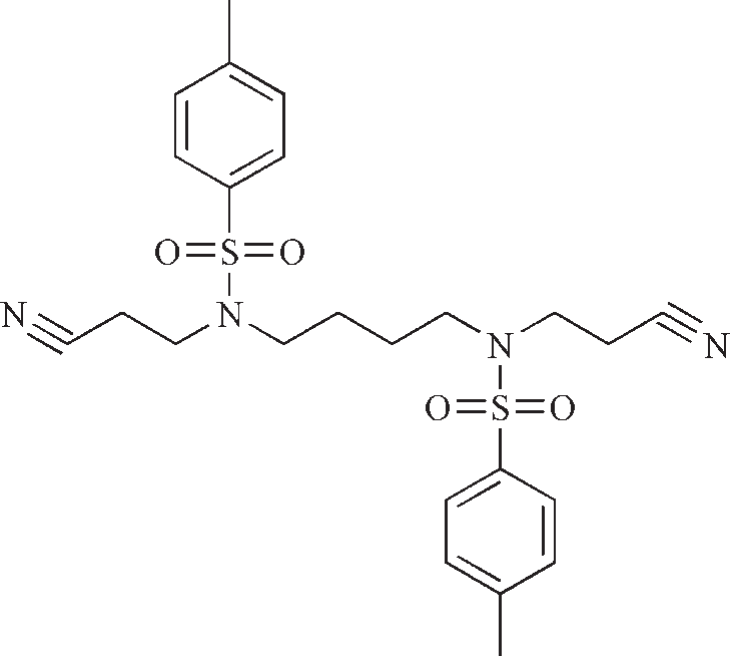


## Experimental

### 

#### Crystal data

C_24_H_30_N_4_O_4_S_2_
*M* *_r_* = 502.64
Monoclinic, 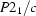

*a* = 16.688 (13) Å
*b* = 5.786 (5) Å
*c* = 13.675 (11) Å
β = 104.005 (13)°
*V* = 1281.3 (17) Å^3^
*Z* = 2
Mo *K*α radiation
μ = 0.25 mm^−1^
*T* = 296 K
0.34 × 0.26 × 0.21 mm

#### Data collection

Bruker SMART CCD diffractometer
Absorption correction: multi-scan (*SADABS*; Sheldrick, 2001 ▶) *T*_min_ = 0.921, *T*_max_ = 0.950
6684 measured reflections
2510 independent reflections
2114 reflections with *I* > 2σ(*I*)
*R*_int_ = 0.019

#### Refinement

*R*[*F* ^2^ > 2σ(*F* ^2^)] = 0.036
*wR*(*F* ^2^) = 0.104
*S* = 1.05
2510 reflections
155 parameters
H-atom parameters constrained
Δρ_max_ = 0.20 e Å^−3^
Δρ_min_ = −0.29 e Å^−3^

### 

Data collection: *SMART* (Bruker, 2001 ▶); cell refinement: *SAINT-Plus* (Bruker, 2001 ▶); data reduction: *SAINT-Plus*; program(s) used to solve structure: *SHELXS97* (Sheldrick, 2008 ▶); program(s) used to refine structure: *SHELXL97* (Sheldrick, 2008 ▶); molecular graphics: *PLATON* (Spek, 2009 ▶); software used to prepare material for publication: *PLATON*.

## Supplementary Material

Crystal structure: contains datablocks global, I. DOI: 10.1107/S1600536809036022/hb5089sup1.cif

Structure factors: contains datablocks I. DOI: 10.1107/S1600536809036022/hb5089Isup2.hkl

Additional supplementary materials:  crystallographic information; 3D view; checkCIF report

## Figures and Tables

**Table 1:** Hydrogen-bond geometry (Å, °)

*D*—H⋯*A*	*D*—H	H⋯*A*	*D*⋯*A*	*D*—H⋯*A*
C5—H5*A*⋯O2^i^	0.93	2.54	3.271 (4)	136

Symmetry code: (i) 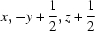.

**Fig. 1.**
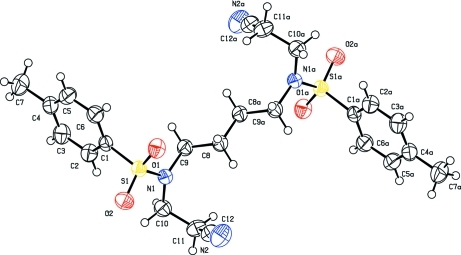
The molecular structure of (I). Displacement ellipsoids are drawn at the 50% probability level. Atoms with the suffix a are generated by (–x, 1–y, 1–z).

**Fig. 2.**
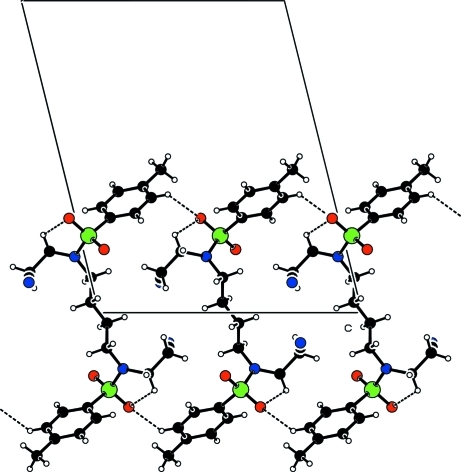
One-dimensional structure of (I) along c axis, Hydrogen bonds are shown as dashed lines.

## supplementary crystallographic information

### Comment

Polyamines are natural products and have interesting biological activities. It present in the majority of cells. They play important roles in the synthesis of proteins, cell division, and bind to nucleic acids resulting in their condensation, thereby affecting gene expression. These effects might have implications in cancer treatment (e.g. Thomas & Thomas, 2003). We now report the crystal structure of the title compound, (I).

As shown in Fig.1, the title compound consists of two 4-methylbenzenesulfonyl groups anchoring to polyamine chain. In the structure of (I), the two phenyl ring of two 4-methylbenzenesulfonyl groups are antiparallel by symmetry.

In the crystal, the molecules are linked into infinite sheets by intermolecular C–H···O hydrogen bonds (Fig. 2).

### Experimental

To a solution of 1,4-diaminobutane (8.8 g, 0.1 mol) in MeOH (300 ml), acrylonitrile (11.66 g, 0.22 mol) was added dropwise at room temperature during 1 h. After stirring for additional 7 h, the solvent was evaporated. The residue was fractionated in vacuum, yielding 17.27 g (89%) of *N*,*N*'-bis(2-cyanoethyl)-1,4-diaminobutane.

To a mixture of *N*,*N*'-bis(2-cyanoethyl)-1,4-diaminobutane (17.27 g, 89 mmol) and Et_3_N (17.98 g, 178 mmol) in THF (120 ml), a solution of 4-methylbenzenesulfonyl chloride (TsCl, 34.02 g, 178 mmol) in THF (120 ml) was added dropwise at room temperature. The precipitate was filtered off and discarded. The filtrate was subsequently washed with 4 mol/*L* NaOH (in order to hydrolyze any unreacted TsCl) and NaCl solution. Evaporation gave the target compound (I) as colourless rods (43.34 g, 97%).

### Refinement

The H atoms were geometrically placed (C—H = 0.93–0.97 Å) and refined as riding with *U*_iso_(*H*) = 1.2*U*_eq_(C) or 1.5*U*_eq_(methyl C).

### Crystal data

**Table:**

C_24_H_30_N_4_O_4_S_2_	*F*(000) = 532
*M**_r_* = 502.64	*D*_x_ = 1.303 Mg m^−^^3^
Monoclinic, *P*2_1_/*c*	Mo *K*α radiation, λ = 0.71073 Å
Hall symbol: -P 2ybc	Cell parameters from 2757 reflections
*a* = 16.688 (13) Å	θ = 3.0–27.6°
*b* = 5.786 (5) Å	µ = 0.25 mm^−^^1^
*c* = 13.675 (11) Å	*T* = 296 K
β = 104.005 (13)°	Rod, colorless
*V* = 1281.3 (17) Å^3^	0.34 × 0.26 × 0.21 mm
*Z* = 2	

### Data collection

**Table:**

Bruker SMART CCD diffractometer	2510 independent reflections
Radiation source: fine-focus sealed tube	2114 reflections with *I* > 2σ(*I*)
graphite	*R*_int_ = 0.019
ω scans	θ_max_ = 26.0°, θ_min_ = 2.5°
Absorption correction: multi-scan (*SADABS*; Sheldrick, 2001)	*h* = −16→20
*T*_min_ = 0.921, *T*_max_ = 0.950	*k* = −7→7
6684 measured reflections	*l* = −16→15

### Refinement

**Table:**

Refinement on *F*^2^	Primary atom site location: structure-invariant direct methods
Least-squares matrix: full	Secondary atom site location: difference Fourier map
*R*[*F*^2^ > 2σ(*F*^2^)] = 0.036	Hydrogen site location: inferred from neighbouring sites
*wR*(*F*^2^) = 0.104	H-atom parameters constrained
*S* = 1.05	*w* = 1/[σ^2^(*F*_o_^2^) + (0.0583*P*)^2^ + 0.2525*P*] where *P* = (*F*_o_^2^ + 2*F*_c_^2^)/3
2510 reflections	(Δ/σ)_max_ < 0.001
155 parameters	Δρ_max_ = 0.20 e Å^−^^3^
0 restraints	Δρ_min_ = −0.28 e Å^−^^3^

### Special details

**Table:**

Geometry. All e.s.d.'s (except the e.s.d. in the dihedral angle between two l.s. planes) are estimated using the full covariance matrix. The cell e.s.d.'s are taken into account individually in the estimation of e.s.d.'s in distances, angles and torsion angles; correlations between e.s.d.'s in cell parameters are only used when they are defined by crystal symmetry. An approximate (isotropic) treatment of cell e.s.d.'s is used for estimating e.s.d.'s involving l.s. planes.
Refinement. Refinement of *F*^2^ against ALL reflections. The weighted *R*-factor *wR* and goodness of fit *S* are based on *F*^2^, conventional *R*-factors *R* are based on *F*, with *F* set to zero for negative *F*^2^. The threshold expression of *F*^2^ > σ(*F*^2^) is used only for calculating *R*-factors(gt) *etc*. and is not relevant to the choice of reflections for refinement. *R*-factors based on *F*^2^ are statistically about twice as large as those based on *F*, and *R*- factors based on ALL data will be even larger.

### Fractional atomic coordinates and isotropic or equivalent isotropic displacement parameters (Å^2^)

**Table:**

	*x*	*y*	*z*	*U*_iso_*/*U*_eq_	
S1	0.24666 (2)	0.32320 (7)	0.53380 (3)	0.03878 (15)	
O1	0.20256 (7)	0.1573 (2)	0.57929 (10)	0.0506 (3)	
O2	0.30224 (8)	0.2415 (2)	0.47571 (10)	0.0558 (3)	
N1	0.17969 (8)	0.4914 (2)	0.46127 (10)	0.0392 (3)	
N2	0.09678 (12)	0.0954 (3)	0.26259 (14)	0.0718 (5)	
C1	0.30449 (9)	0.4988 (3)	0.63262 (12)	0.0371 (4)	
C2	0.33821 (10)	0.7065 (3)	0.61032 (13)	0.0469 (4)	
H2A	0.3275	0.7592	0.5442	0.056*	
C3	0.38797 (11)	0.8344 (3)	0.68757 (15)	0.0532 (5)	
H3A	0.4108	0.9727	0.6725	0.064*	
C4	0.40431 (10)	0.7595 (4)	0.78710 (14)	0.0496 (4)	
C5	0.36879 (11)	0.5538 (4)	0.80728 (13)	0.0541 (5)	
H5A	0.3787	0.5020	0.8735	0.065*	
C6	0.31883 (11)	0.4232 (3)	0.73143 (13)	0.0498 (4)	
H6A	0.2952	0.2865	0.7468	0.060*	
C7	0.45863 (13)	0.8996 (4)	0.87086 (16)	0.0720 (6)	
H7A	0.4839	1.0233	0.8423	0.108*	
H7B	0.5008	0.8020	0.9103	0.108*	
H7C	0.4257	0.9625	0.9131	0.108*	
C8	0.03371 (9)	0.4433 (3)	0.47933 (13)	0.0450 (4)	
H8A	0.0144	0.4254	0.4069	0.054*	
H8B	0.0455	0.2908	0.5087	0.054*	
C9	0.11282 (9)	0.5880 (3)	0.50313 (12)	0.0429 (4)	
H9A	0.0999	0.7425	0.4765	0.052*	
H9B	0.1326	0.6008	0.5757	0.052*	
C10	0.19632 (11)	0.5953 (3)	0.37044 (13)	0.0469 (4)	
H10A	0.2493	0.5407	0.3622	0.056*	
H10B	0.1997	0.7618	0.3785	0.056*	
C11	0.12903 (12)	0.5356 (3)	0.27550 (12)	0.0497 (4)	
H11A	0.0788	0.6183	0.2772	0.060*	
H11B	0.1466	0.5868	0.2164	0.060*	
C12	0.11145 (12)	0.2864 (4)	0.26701 (13)	0.0499 (4)	

### Atomic displacement parameters (Å^2^)

**Table:**

	*U*^11^	*U*^22^	*U*^33^	*U*^12^	*U*^13^	*U*^23^
S1	0.0360 (2)	0.0370 (3)	0.0428 (2)	0.00198 (15)	0.00858 (17)	−0.00311 (16)
O1	0.0517 (7)	0.0410 (7)	0.0564 (7)	−0.0076 (5)	0.0077 (6)	0.0045 (5)
O2	0.0506 (7)	0.0601 (8)	0.0597 (7)	0.0117 (6)	0.0191 (6)	−0.0116 (7)
N1	0.0341 (7)	0.0462 (8)	0.0371 (7)	0.0025 (6)	0.0086 (5)	−0.0004 (6)
N2	0.0825 (13)	0.0529 (11)	0.0736 (12)	−0.0051 (10)	0.0062 (10)	−0.0087 (9)
C1	0.0296 (7)	0.0394 (9)	0.0420 (8)	0.0018 (6)	0.0080 (6)	−0.0010 (7)
C2	0.0475 (9)	0.0444 (10)	0.0470 (9)	−0.0020 (8)	0.0075 (8)	0.0057 (8)
C3	0.0520 (10)	0.0429 (10)	0.0627 (12)	−0.0099 (8)	0.0100 (9)	−0.0004 (8)
C4	0.0377 (9)	0.0570 (11)	0.0531 (10)	−0.0020 (8)	0.0092 (8)	−0.0103 (9)
C5	0.0539 (10)	0.0667 (13)	0.0400 (9)	−0.0098 (9)	0.0078 (8)	−0.0006 (9)
C6	0.0490 (10)	0.0542 (11)	0.0464 (9)	−0.0109 (8)	0.0118 (8)	0.0035 (8)
C7	0.0594 (12)	0.0813 (16)	0.0688 (14)	−0.0156 (11)	0.0033 (10)	−0.0215 (12)
C8	0.0369 (8)	0.0532 (11)	0.0440 (9)	0.0028 (8)	0.0078 (7)	−0.0079 (8)
C9	0.0371 (8)	0.0465 (10)	0.0441 (9)	0.0045 (7)	0.0077 (7)	−0.0060 (8)
C10	0.0493 (10)	0.0462 (10)	0.0465 (9)	−0.0050 (8)	0.0140 (8)	0.0028 (8)
C11	0.0633 (11)	0.0454 (10)	0.0388 (9)	0.0042 (8)	0.0092 (8)	0.0056 (7)
C12	0.0549 (11)	0.0527 (12)	0.0395 (9)	0.0032 (9)	0.0062 (8)	−0.0036 (8)

### Geometric parameters (Å, °)

**Table:**

S1—O1	1.4392 (14)	C6—H6A	0.9300
S1—O2	1.4393 (14)	C7—H7A	0.9600
S1—N1	1.6259 (15)	C7—H7B	0.9600
S1—C1	1.7772 (18)	C7—H7C	0.9600
N1—C10	1.466 (2)	C8—C8^i^	1.524 (3)
N1—C9	1.481 (2)	C8—C9	1.530 (2)
N2—C12	1.130 (3)	C8—H8A	0.9700
C1—C6	1.385 (2)	C8—H8B	0.9700
C1—C2	1.392 (2)	C9—H9A	0.9700
C2—C3	1.389 (3)	C9—H9B	0.9700
C2—H2A	0.9300	C10—C11	1.536 (2)
C3—C4	1.391 (3)	C10—H10A	0.9700
C3—H3A	0.9300	C10—H10B	0.9700
C4—C5	1.387 (3)	C11—C12	1.471 (3)
C4—C7	1.513 (3)	C11—H11A	0.9700
C5—C6	1.387 (3)	C11—H11B	0.9700
C5—H5A	0.9300		
			
O1—S1—O2	118.99 (9)	H7A—C7—H7B	109.5
O1—S1—N1	108.36 (9)	C4—C7—H7C	109.5
O2—S1—N1	107.44 (9)	H7A—C7—H7C	109.5
O1—S1—C1	107.02 (9)	H7B—C7—H7C	109.5
O2—S1—C1	107.68 (9)	C8^i^—C8—C9	111.23 (18)
N1—S1—C1	106.75 (9)	C8^i^—C8—H8A	109.4
C10—N1—C9	119.19 (14)	C9—C8—H8A	109.4
C10—N1—S1	121.28 (12)	C8^i^—C8—H8B	109.4
C9—N1—S1	117.45 (11)	C9—C8—H8B	109.4
C6—C1—C2	120.19 (16)	H8A—C8—H8B	108.0
C6—C1—S1	119.69 (14)	N1—C9—C8	113.80 (14)
C2—C1—S1	120.06 (13)	N1—C9—H9A	108.8
C3—C2—C1	119.52 (17)	C8—C9—H9A	108.8
C3—C2—H2A	120.2	N1—C9—H9B	108.8
C1—C2—H2A	120.2	C8—C9—H9B	108.8
C2—C3—C4	121.16 (18)	H9A—C9—H9B	107.7
C2—C3—H3A	119.4	N1—C10—C11	111.99 (15)
C4—C3—H3A	119.4	N1—C10—H10A	109.2
C5—C4—C3	118.05 (17)	C11—C10—H10A	109.2
C5—C4—C7	121.07 (18)	N1—C10—H10B	109.2
C3—C4—C7	120.88 (19)	C11—C10—H10B	109.2
C4—C5—C6	121.83 (17)	H10A—C10—H10B	107.9
C4—C5—H5A	119.1	C12—C11—C10	112.22 (15)
C6—C5—H5A	119.1	C12—C11—H11A	109.2
C1—C6—C5	119.23 (18)	C10—C11—H11A	109.2
C1—C6—H6A	120.4	C12—C11—H11B	109.2
C5—C6—H6A	120.4	C10—C11—H11B	109.2
C4—C7—H7A	109.5	H11A—C11—H11B	107.9
C4—C7—H7B	109.5	N2—C12—C11	178.0 (2)
			
O1—S1—N1—C10	−149.35 (13)	C1—C2—C3—C4	−0.4 (3)
O2—S1—N1—C10	−19.60 (15)	C2—C3—C4—C5	−0.6 (3)
C1—S1—N1—C10	95.68 (14)	C2—C3—C4—C7	179.95 (17)
O1—S1—N1—C9	47.17 (14)	C3—C4—C5—C6	0.5 (3)
O2—S1—N1—C9	176.93 (11)	C7—C4—C5—C6	179.98 (18)
C1—S1—N1—C9	−67.79 (13)	C2—C1—C6—C5	−1.6 (3)
O1—S1—C1—C6	17.62 (16)	S1—C1—C6—C5	175.56 (13)
O2—S1—C1—C6	−111.40 (15)	C4—C5—C6—C1	0.6 (3)
N1—S1—C1—C6	133.48 (14)	C10—N1—C9—C8	102.87 (18)
O1—S1—C1—C2	−165.17 (13)	S1—N1—C9—C8	−93.29 (15)
O2—S1—C1—C2	65.80 (15)	C8^i^—C8—C9—N1	−177.68 (17)
N1—S1—C1—C2	−49.32 (15)	C9—N1—C10—C11	−73.0 (2)
C6—C1—C2—C3	1.6 (3)	S1—N1—C10—C11	123.77 (15)
S1—C1—C2—C3	−175.62 (13)	N1—C10—C11—C12	−50.0 (2)

Symmetry codes: (i) −*x*, −*y*+1, −*z*+1.

### Hydrogen-bond geometry (Å, °)

**Table:**

*D*—H···*A*	*D*—H	H···*A*	*D*···*A*	*D*—H···*A*
C5—H5A···O2^ii^	0.93	2.54	3.271 (4)	136

Symmetry codes: (ii) *x*, −*y*+1/2, *z*+1/2.
